# Automated machine learning in nanotoxicity assessment: A comparative study of predictive model performance

**DOI:** 10.1016/j.csbj.2024.02.003

**Published:** 2024-02-09

**Authors:** Xiao Xiao, Tung X. Trinh, Zayakhuu Gerelkhuu, Eunyong Ha, Tae Hyun Yoon

**Affiliations:** a Department of Chemistry, College of Natural Sciences, Hanyang University, Seoul 04763, the Republic of Korea; b Institute of Next Generation Material Design, Hanyang University, Seoul 04763, the Republic of Korea; c Yoon Idea Lab. Co. Ltd, Seoul 04763, the Republic of Korea

**Keywords:** Nanotoxicity modeling, Automated machine learning, Oxide, Metal, Data quality

## Abstract

Computational modeling has earned significant interest as an alternative to animal testing of toxicity assessment. However, the process of selecting an appropriate algorithm and fine-tuning hyperparameters for the developing of optimized models takes considerable time, expertise, and an intensive search. The recent emergence of automated machine learning (autoML) approaches, available as user-friendly platforms, has proven beneficial for individuals with limited knowledge in ML-based predictive model development. These autoML platforms automate crucial steps in model development, including data preprocessing, algorithm selection, and hyperparameter tuning. In this study, we used seven previously published and publicly available datasets for oxides and metals to develop nanotoxicity prediction models. AutoML platforms, namely Vertex AI, Azure, and Dataiku, were employed and performance measures such as accuracy, F1 score, precision, and recall for these autoML-based models were then compared with those of conventional ML-based models. The results demonstrated clearly that the autoML platforms produced more reliable nanotoxicity prediction models, outperforming those built with conventional ML algorithms. While none of the three autoML platforms significantly outperformed the others, distinctions exist among them in terms of the available options for choosing technical features throughout the model development steps. This allows users to select an autoML platform that aligns with their knowledge of predictive model development and its technical features. Additionally, prediction models constructed from datasets with better data quality displayed, enhanced performance than those built from datasets with lower data quality, indicating that future studies with high-quality datasets can further improve the performance of those autoML-based prediction models.

## Graphical Abstract

**Figure:**
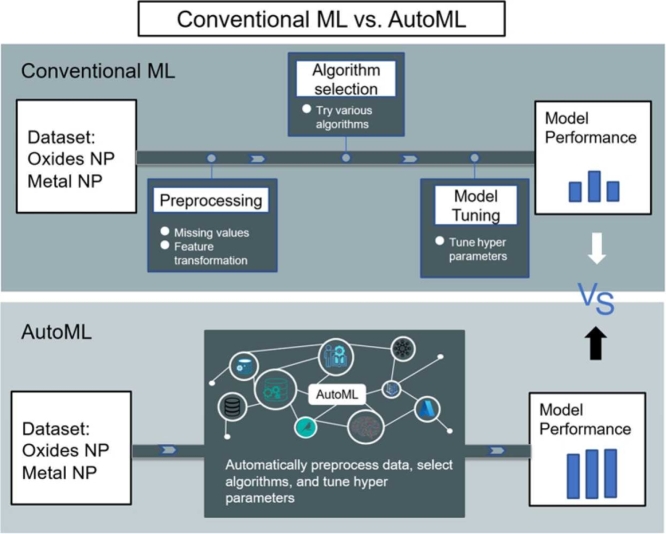

## 1 Introduction

Nanomaterials, characterized by at least one dimension within the range of 1–100 nm, find diverse applications in consumer products and industries, including cosmetics [1], photocatalytic materials [2], bio-sensing [3], and drug delivery [4]. With a wide range of applications, nanomaterials are used in various nanoproducts with potential risks of exposure to humans and the environment. Due to their increased use, both intended and unintended exposure may occur in living organisms, leading to potential hazards and adverse outcomes. Recent studies on the toxic effects of nanomaterials on the environment and biological systems have shown that nanomaterials can cause adverse outcomes in cultured cells [5], aquatic organisms [6], and mammalians [7]. A comprehensive toxicity assessment of nanomaterials is important for manufacturers of nanoproducts. Regulatory frameworks, such as the registration, evaluation, authorization, and restriction of chemicals (REACH), require manufacturers to evaluate the safety of nanomaterials and nano-products before release to the European market [8]. Traditionally, toxicity tests of chemicals, including nanomaterials, were conducted using animals (in vivo) and cultured cells (in vitro), which are expensive and time-consuming. In response to the 3 R strategy-reduction, reﬁnement, and replacement aimed at minimizing the use of animals in biological research [9], alternative approaches to hazard assessment, such as in silico methods, have recently attracted significant attention. Furthermore, the number of studies on nanotoxicity has increased with data systematically curated for non-animal nanotoxicity tests [10], [11], [12], [13], [14], [15], [16]. The need for high-throughput and low-cost screening methods has led to the rapid development of computational modeling of nanotoxicity, particularly with machine learning (ML) models [17], [18], [19], [20], [21], [22].

To develop ML models for nanotoxicity prediction, physicochemical and toxicological data of nanomaterials were used. Specifically, endpoints, representing toxic effects on living organisms following exposure to nanomaterials, were chosen as dependent variables. In contrast, the descriptors, comprising the physicochemical and theoretical properties of nanomaterials, along with exposure parameters, were designated as independent variables for the models. The relationship between these endpoints and descriptors is discerned by ML algorithms employing various types of mathematical functions. To build a satisfactory prediction model, it is necessary to perform data preprocessing, algorithm selection, and hyperparameter tuning, which requires time, expertise, and computing power for an intensive search of optimized parameters.

Recently, a new modeling tool, automated machine learning (autoML), has attracted the attention of researchers across various fields [23]. With autoML, the entire ML pipeline, including data preprocessing, selection of suitable algorithms, and hyper-parameter tuning, is merged and automated [24], [25]. AutoML returns an optimal model by comparing the performance of various candidates. A complete autoML workflow is considered able to create an easy-to-use and end-to-end ML pipeline system through a dynamic combination of multiple processes and algorithms. Many autoML systems are now available as a result of recent advancements in this field including Auto-sklearn [26], Auto-keras [27], H_2_O-autoML [28], and Tree-based Pipeline Optimization Tool [29]. However, it is important to note that these tools still require some knowledge of coding and programming. More recently, several artificial intelligence (AI) companies have developed publicly available autoML platforms such as Vertex AI [30], Microsoft Azure [31], and Dataiku [32], aiming to assist people with little or no ML expertise in creating high-performance custom models within a short time [33]. These platforms minimize the need for decisions regarding technical features. Despite the widespread availability and accessibility of autoML platforms, their application in the field of nanosafety research is rare, except for the recent case involving superparamagnetic iron oxide nanoparticles [34]. Here, Kotzabasaki et al. curated the nanotoxicity data of 16 iron oxide nanoparticles from 12 publications and developed models predicting the cellular toxicity of these nanoparticles using the autoML platform named Tree-based Pipeline Optimization Tool [29].

In this study, to benefit the users with limited knowledge about ML and predictive model developments, we employed user-friendly autoML platforms, such as Vertex AI [30], Azure [31], and Dataiku [32]. These platforms are equipped with graphical user interfaces and require minimal technical decisions regarding features. To assess the feasibility of these autoML platforms in the field of nanosafety research, we developed nanotoxicity prediction models based on autoML using publicly available datasets [11], [12], and compared their performance to that of conventional ML algorithm based models. In addition to the comparison with conventional ML-based models, we explore the effects of data quality and dataset size on model performance. This comprehensive analysis aim to shed light on the suitability of autoML platforms for nanosafety research and provides insights into their comparative advantages.

## 2 Materials & methods

### 2.1 Workflow

**Fig. 1:**
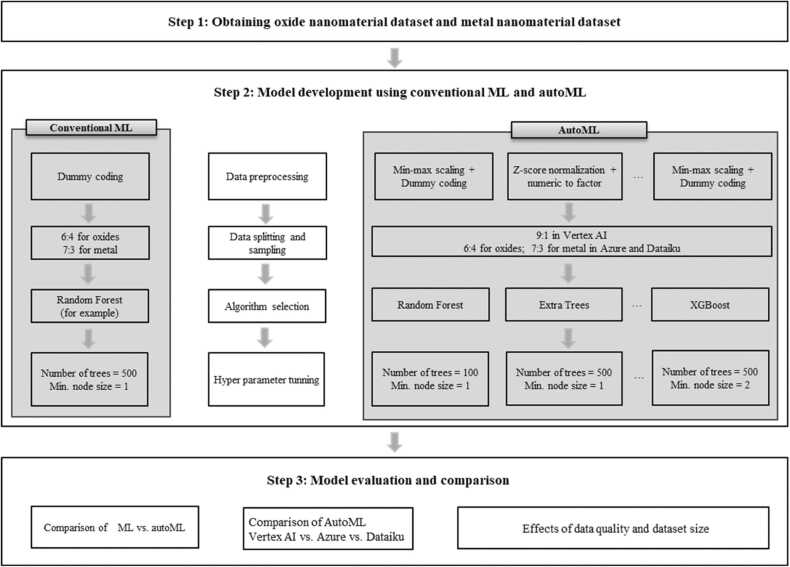
Workflow of data collection (Step 1), model development (Step 2), and model evaluation and comparison (Step 3).

The workflow of this study consists of three steps: dataset collection, prediction model development, and model evaluation and comparison (Fig. 1). Firstly, publicly available nanotoxicity datasets for metal and oxide nanoparticles were collected from literature, which were curated from 216 publications on oxide nanoparticles and 63 publications on metal nanoparticles [11], [12]. Secondly, nanotoxicity prediction models were developed based on two different approaches, such as conventional ML algorithms and autoML platforms. Previously, classification models were developed from these datasets using conventional ML algorithms (e.g., random forest, support vector machine, and gradiaent boosting tree). Along with these ML algorithms, three autoML platforms were used to automate development steps of nanotoxicity prediction models. Thirdly, models developed using both approaches were compared based on their performance measures such as accuracy, F1 score, precision, and recall.

### 2.2 Datasets

Seven publicly available datasets were collected and used directly for model development without any modification. Four of them are datasets for oxide nanomaterials and are denoted as Ha I, Ha II, Ha IIIA, and Ha IIIB. These oxide datasets were collected from 216 published articles, which contain the cytotoxicity of 26 metal oxide NPs to various cell lines. As described in Table 1, they are comprised of 17 columns with 6842, 3246, 1738, and 666 rows, respectively. The other three are datasets for metal nanomaterials and are denoted as Trinh A, Trinh B, and Trinh C. These three datasets were collected from 63 published articles, which contain the cytotoxicity of gold and silver NPs to various cell lines. All these metal datasets are comprised of 16 columns with 2005 rows.

**Table 1:** Dataset description.

Material type	Dataset name	Source	No. rows	No. descriptors	PChem Score	Dataset description	Descriptors	Ref.
Oxide	Ha Ⅰ	S2Nano: 216 articles	6842	15	2.8 ± 1.2	Raw data collected from literature	Core size, hydrodynamic size, surface charge, surface area, ΔH_sf_, E_c_, E_v_, χ^Meo^, assay, cell name, cell species, cell origin, cell type, exposure time, exposure dose.	Ha et al.[11]
	Ha Ⅱ	S2Nano: filtered from Ha I	3246	15	4.7 ± 0.2	Gap filled and filtered from Ha Ⅰ		
	Ha ⅢA	S2Nano: filtered from Ha II	1738	15	4.8 ± 0.1	50% rows from Ha Ⅱ with highest PChem scores		
	Ha ⅢB	S2Nano: filtered from Ha II	666	15	4.9 ± 0.1	20% rows from Ha Ⅱ with highest PChem scores		
Metal	Trinh A	S2Nano: 63 articles	2005	14	4.3 ± 0.4	Data collected from literature and gap filled	Core size, hydrodynamic size, surface charge, specific surface area, shape, coating, metal type, dose, assay, time, species, cancer, cell tissue, cell line.	Trinh et al.[12]
	Trinh B	S2Nano: 63 articles	2005	14	4.1 ± 0.4	Replace all experimental data in Trinh A with manufacturer information		
	Trinh C	S2Nano: 63 articles	2005	14	2.9 ± 1.9	Replace all non-experimental data in Trinh B with mean values of experimental data		

In these datasets, we implemented an innovative technique for addressing missing values, known as data gap filling [11], [12]. This method involved substituting the missing physicochemical attributes of target nanoparticles with corresponding data from source NPs that were theoretically similar in properties. For cases where well-documented commercial materials had missing data, we used the respective manufacturers’ characterization data for NPs that shared the same brand and product number. This was based on the presumption that NPs produced by the same manufacturer would possess comparable properties. In instances of gaps in specific surface area data, we employed a calculation based on core size, and vice versa. The formula used was SSA = 6/(d × ρ), where SSA signifies the specific surface area, d is the diameter, and ρ represents the density of the nanoparticle. For missing quantum mechanical properties, we utilized replacement data extracted from the research findings of Zhang et al., Liu et al. [35], [36]. Data quality was assessed with PChem score, which we previously developed and proposed as criteria for estimating the physicochemical data's quality of nanomaterials [11], [12]. The PChem score criteria are determined by two factors: the source of data and the method of measurement. Regarding the source of data, there are four categories: data that contain no information on the source of data, data that were adapted from other references using the same nanomaterial, data that were adapted from manufacturer's specification, and data that were measured from the authors own experiments. Each category is scored as 0, 1, 2, 3, respectively. As for the measurement method, there are three categories: data that contain no information on method used, data that were measured using non-standard methods, and data that were measured using standard measurement methods. The scores for those three categories are denoted by the numbers 0, 1, 2, respectively. Based on these criteria, each row will have four score values that correspond to the physicochemical properties of core size, hydrodynamic size, surface charge, and surface area. The score for each row is calculated by averaging these four values. The PChem score for each dataset is determined as the average of the scores for each row. The PChem scores for the seven dataset used in this study were listed in Table 1.

### 2.3 Model development

ML algorithms and autoML platforms were used to construct models predicting the cellular toxicity (toxic or nontoxic) of metal and oxide nanoparticles. The endpoint is the toxicity (toxic or nontoxic), which is based on cell viability (cell viability less than 50% is toxic, and cell viability greater than 50% is nontoxic). The criteria for toxic/nontoxic classification could be variable as the experimental conditions (e.g., dose, types of nanomaterials, and cell types) changes. However, as suggested by the OECD (Organization for Economic Co-operation and Development) principle of (Q)SAR (quantitative structure-activity relation) validation [37], we have defined endpoint with the above criteria to ensure fair and robust comparisons, which was previously chosen and used for the earlier studies [11], [12]. The modeling workflow for conventional ML algorithms and autoML platforms is illustrated in Fig. 1.

For the developments of ML-based models, dummy encoding was used for the categorical descriptors. This step uses N-1 binary values to encode one categorical value with N categories. For example, the Trinh A dataset has a categorical descriptor “shape” with three types (nanorod, sphere, and hollow). These three categories were replaced with two dummy variables (nanorod: 01, sphere: 10, and hollow: 00). Random forest [38], support vector machine [39], and gradient-boosted tree [40] algorithms were used to develop the conventional ML models. Following the methods in the previously published studies, the oxide datasets were split into a 60% training set and a 40% test set, and the metal datasets were split into a 70% training set and a 30% test set. A stratified sampling method was used, which keeps the ratio of toxic/nontoxic data in the training set to the same ratio as in the test set. Trained models were developed on the training set using the above algorithms, and the test set was used to validate the models. A 10-fold cross-validation was applied to the training datasets to avoid overfitting. RapidMiner Studio Educational version 9.8.000 (https://rapidminer.com/) was used to develop the models. With the random forest algorithm, the number of trees (number of random trees to generate) was set to 500. The minimum node size (minimum number of data points required to be at a leaf node) was set to 1. For the support vector machine algorithm, we used a dot kernel (the kernel function defined by the inner product of two points), the C value (SVM complexity constant which sets the tolerance for misclassification) of which was 1.0. For the gradient boosted trees algorithm, the number of trees, learning rate (a parameter used to control the weighting of new trees added to the model), and maximum tree depth (a measure of how many splits a tree can make before coming to a prediction) were 50, 0.1, and 5, respectively.

For the developments of autoML-based models, the Vertex AI [30], Azure [31], and Dataiku [32] were selected. In these autoML platforms, after uploading datasets, users can develop models with the default options of pipeline or users can have more control over the modelling process, via modifying certain parameters. In the Vertex AI [30], the dataset was uploaded through “Vertex AI - datasets - create” menu and the prediction model was developed through the “Vertex AI - models - create” menu. In Vertex AI, the dataset must have a minimum of 1000 rows; otherwise, model training will not proceed. The datasets were randomly splitted into the training, test, and validation sets with split ratio of 0.8, 0.1, 0.1, respectively. This ratio can not be user modified in Vertex AI, whereas the other platforms allow for adjusting this split ratio. Currently, Vertex AI platform does not support cross-validation so the training sets were randomly split into two subsets, one for training the model and one for tuning hyperparameters. The optimization objective was the area under the receiver operating characteristic curve (AUC ROC). About 1 hr is required to train the autoML model for a tabular dataset of thousands of rows and tens of columns. During the training time, Vertex AI trained a model called AutoML classification which utilizes algorithms like Boosted Tree and Artificial Neural Network. When the training was finished, the platform provided the performance of the best model in the “model” section, where we were able to check the confusion matrix and descriptor importance. The parameters of the best model and the other trained models are available in the “model properties” section.

In the Azure [31] environment, the autoML model development took place under the sequence “machine learning – automated ML – start run”. In “configure run – view additional configuration settings”, the split ratio of the training set and test set was set as 6:4 for oxide dataset and 7:3 for metal dataset. The split was random, to prevent overfitting, the training sets were subjected to a 5-fold cross-validation which is not available on Vertex AI. The optimization objective was the weighted AUC. On average, approximately 50 models which utilized ensemble or individual algorithms were generated, and a summary of the models was provided, along with the best model performance, hyperparameters, and descriptor importance. In Dataiku [32], the autoML development was in “Lab - AutoML prediction”. In the “design - training/test set” panel, the split ratio of training set and test set was set to 6:4 for oxide dataset and 7:3 for metal dataset. The split was random. A 5-fold cross-validation was applied to the training sets to avoid overfitting. The modeling hyperparameters were optimized for the F1 score. In the “design - modeling - algorithms” panel, we chose all 13 algorithms provided on this website and used the default ranges of hyperparameters for all algorithms. It took approximately 20 min to build the autoML model for one dataset of the above-mentioned size. The tuned hyperparameters for each model, as well as a confusion matrix and descriptor importance, were in the “analysis-models” section.

We have also built ML and autoML models, with the same splitting of training and test sets for a particular dataset. We assessed whether there were activity cliffs (unexpected large 'jumps' in activity due to small changes in structure) in these test sets using the Banerjee and Roy Similarity Coefficients 1 and 2 (S_m_^1^ and S_m_^2^)[41]. Briefly, for each row of the test set, its similarity values with respect to each row in the training set was calculated. Then, the similarity values of the closest positive source nanomaterial, the closest negative source nanomaterial, the average similarity values of the positive and negative close source nanomaterials were derived and plugged into the equations for calculating the S_m_^1^ and S_m_^2^.

### 2.4 Model evaluation and comparison

The model performance was evaluated by using the F1 scores, accuracy, precision, and recall derived from the confusion matrix [42]. These four ML metrics were compared to those metrics of autoML to determine whether autoML performs better than ML. To better understand the differences in the model performance between the two approaches, we investigated the autoML workflow to see how the models were developed inside the autoML platforms. A comparison among the different autoML platforms was conducted to determine whether one platform outperformed the others. Because our datasets were preprocessed to have a different quality and size, we also discussed the effects of the quality and size on the performance of the autoML and ML models.

## 3 Results and discussion

### 3.1 Datasets used for model development

As summarized in Table 1, two groups of datasets were used in this study. The first group is made up of datasets for oxide nanomaterials, which are labeled as Ha I, II, IIIA, and IIIB [12]. Fifteen descriptors were used for model development in this group of datasets, including physicochemical properties (e.g., core size, hydrodynamic size, surface charge, and specific surface area), biological properties related to in vitro toxicity test (e.g., assay, cell name, cell species, cell origin, cell type, exposure time, and exposure dose), and some quantum-mechanical properties (e.g., enthalpy of formation (ΔH_sf_), conduction band (E_c_), valence band (E_v_), and electronegativity (χ^Meo^)), which have been previously reported as important parameters related to nanotoxicity [36], [43]. These datasets for oxide nanomaterials were originally designed to test the effects of data quality and dataset size. The raw data collected from literature (i.e., Ha I) were preprocessed to improve data quality, sorted according to the quality of each row (i.e., PChem score), and selected top 100% (i.e., Ha II), 50% (i.e., Ha IIIA), and 20% (i.e., Ha IIIB) rows. As shown in Table 1, the Ha I, II, IIIA, and IIIB datasets contain decreasing numbers of rows (6842, 3246, 1738, and 666, respectively) with increasing quality (PChem score of 2.8 ± 1.2, 4.7 ± 0.2, 4.8 ± 0.1, and 4.9 ± 0.1, respectively). The datasets for metallic nanoparticles, labeled as Trinh A, B, and C, make up the second group [11]. The datasets in the second group are structured similarly to those in the first group, but slightly different. In this group of datasets, biological parameters as well as physicochemical characteristics were used as descriptors, but quantum mechanical properties are not included in the list of descriptors. These datasets for metallic nanomaterials were originally designed to test the data quality effects on the datasets with the same size. As shown in Table 1, the Trinh A, B, and C datasets have the same dataset size (numbers of rows, 2005) with decreasing data quality (PChem score of 4.3 ± 0.4, 4.1 ± 0.4, and 2.9 ± 1.9, respectively). Further details on each dataset's descriptor of were analyzed and illustrated in Fig. S1-S7, which contains three plots corresponding to the distributions of key physicochemical properties, dose-viability relationship, and material types/cell species included in the datasets. The distributions of core size, hydrodynamic size, surface charge, and specific surface area were illustrated in the first plot of each figure, while the scatter plots of dose-viability data and pie chart on the distribution of material types and cell species were given as the second and third plots, respectively.

There were some activity cliffs presented by S_m_^1^ and S_m_^2^ values (Fig. S8), indicating that small fluctuations in the features could cause changes in nanotoxicity.

### 3.2 Comparison of model performances: conventional ML vs. autoML

**Fig. 2:**
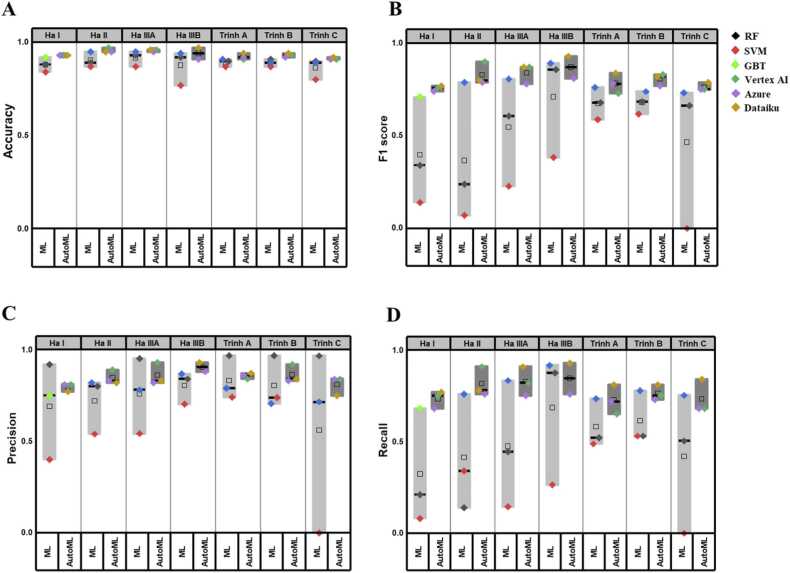
Accuracy (A), F1 score (B), precision (C), and recall (D) of ML and autoML models on datasets Ha I, II, IIIA, and IIIB, and Trinh A, B, C. In the boxplot each dot represents one algorithm, the black line is the median and the square is the mean. RF: random forest. SVM: support vector machine. GBT: gradient boosted trees.

The performance measures of conventional ML and autoML based models (i.e., accuracy, F1 score, precision, and recall) were calculated for each dataset and are provided in Table 2. Based on these values, the performances of conventional ML and autoML based models were plotted in Fig. 2. Overall, the performances of autoML based models displayed higher mean values with smaller deviations. As shown in Table 2 and Fig. 2A, for the conventional ML based models built with seven datasets, the mean values of accuracy ranged between 0.80 and 0.92 with standard deviations of 0.02 to 0.04, while those of autoML based models for each dataset were found slightly improved, ranging from 0.91 to 0.96 with standard deviations of 0.01 to 0.03. As previously described by Ha et al. [12] and Trinh et al. [11], the datasets used in this study also have the imbalance issues, where the majority of the data rows belonged to the nontoxic class. Although considered as one of the most commonly used measure for evaluating classification models, the accuracy could be sometimes misleading, particularly for the classification of imbalanced datasets. These imbalances in the datasets make the predictions biased to the dominant (i.e., nontoxic) class and might impair their generalization. Therefore, to correctly evaluate the model performance, the F1 score, defined as the harmonic mean of precision and recall, could be more suitable measure than the accuracy. As shown in Table 2 and Fig. 2B, the mean F1 scores of autoML-based models for each dataset ranged between 0.76 and 0.87 with standard deviations of 0.01 to 0.06, whereas those of conventional ML-based models ranged between 0.29 and 0.78 with standard deviations of 0.06 to 0.23. Compared to the corresponding accuracy values, the F1 scores of conventional ML-based models displayed much lower mean values with wider variations, while the F1 scores of autoML-based models displayed significantly improved performances compared to those of ML based models, with much higher mean values and smaller variations. Similar with the F1 score, as shown in Fig. 2C,D, precision (positive predictive value) and recall (sensitivity) displayed lower mean values with larger variations for the conventional ML-based models. Conversely, significant increments of their mean values were observed for the autoML-based models, accompanied by dramatic reductions of their variations. When the precision of a nanotoxicity prediction model is low, some non-toxic, safe nanomaterials could be predicted as toxic and considered as harmful, so limiting their potential uses in various industrial applications. In contrast, if the recall of nanotoxicity prediction models is low, potentially hazardous nanoparticles could be predicted as non-toxic, posing a serious health risk to consumers. Therefore, although precision and recall are inversely related [41], high precision and recall are desirable properties for reliable prediction models. The improvements of all the four performance measures with remarkable reductions in their variations demonstrates that autoML platforms delivers more reliable and better performing models than conventional ML algorithms. The advantage of using autoML for modelling was also observed (Fig. S9), when we used the same training and test sets for a particular data set. Moreover, as described earlier, the seven datasets used in this study include experimental data on various nanomaterials measured in different experimental settings, such as assay methods, cell lines, doses, and exposure times. Table S1 provides a comparison of experimental and predicted toxicity values for several well-known nanomaterials, as sample data for the various nanomaterials measured in different experimental settings. For those nanomaterials reported with experimentally "non-toxic" values under the various exposure conditions given in the literature, both conventional ML and autoML-based models perform similarly well (e.g., Al_2_O_3_, CeO_2_, TiO_2_ and Au). However, for those reported as "toxic" under the experimental conditions (e.g., ZnO and Ag), autoML-based models outperform models built with conventional ML algorithms. These observations of significant improvements in the autoML-based model performances for all these datasets demonstrates that the nanotoxicity models developed with autoML platforms are performing well irrespective of the diversity in material types and experimental settings.

**Table 2:** Performance of conventional ML and autoML models. RF: random forest, SVM: support vector machine, and GBT: gradient boosted trees.

			Oxide					Metal				
		Algorithm / Platform	Ha I	Ha II	Ha IIIA	Ha IIIB	Trinh A		Trinh B	Trinh C	Mean ± SD	
Accuracy	ML	RF	0.88	0.89	0.93	0.92	0.90		0.91	0.9	0.90 ± 0.02	
		SVM	0.84	0.87	0.87	0.77	0.87		0.87	0.80	0.804 ± 0.04	
		GBT	0.92	0.95	0.95	0.94	0.91		0.89	0.89	0.92 ± 0.02	
		Mean ± SD	0.88 ± 0.03	0.90 ± 0.03	0.92 ± 0.03	0.88 ± 0.08	0.89 ± 0.02		0.89 ± 0.02	0.86 ± 0.05		
	AutoML	Vertex AI	0.93	0.97	0.96	--	0.91		0.93	0.91	0.94 ± 0.02	
		Azure	0.93	0.95	0.95	0.91	0.92		0.92	0.91	0.93 ± 0.03	
		Dataiku	0.93	0.95	0.96	0.97	0.94		0.94	0.92	0.94 ± 0.02	
		Mean ± SD	0.93 ± 0.00	0.96 ± 0.01	0.96 ± 0.01	0.94 ± 0.03	0.92 ± 0.01		0.93 ± 0.01	0.91 ± 0.01		
F1 score	ML	RF	0.34	0.24	0.61	0.86	0.68		0.69	0.66	0.58 ± 0.20	
		SVM	0.14	0.07	0.23	0.38	0.59		0.62	0.00	0.29 ± 0.23	
		GBT	0.71	0.79	0.81	0.89	0.76		0.74	0.73	0.78 ± 0.06	
	Mean ± SD		0.40 ± 0.24	0.37 ± 0.31	0.55 ± 0.24	0.71 ± 0.23	0.68 ± 0.07		0.68 ± 0.05	0.46 ± 0.33		
	AutoML	Vertex AI	0.76	0.909	0.87	--	0.73		0.83	0.75	0.81 ± 0.06	
		Azure	0.74	0.79	0.78	0.81	0.78		0.77	0.75	0.77 ± 0.02	
		Dataiku	0.77	0.80	0.87	0.93	0.84		0.82	0.79	0.83 ± 0.05	
	Mean ± SD		0.76 ± 0.01	0.83 ± 0.05	0.84 ± 0.04	0.87 ± 0.06	0.78 ± 0.05		0.81 ± 0.03	0.76 ± 0.02		
Precision	ML	RF	0.92	0.80	0.95	0.84	0.97		0.97	0.97	0.92 ± 0.07	
		SVM	0.40	0.54	0.54	0.70	0.74		0.74	0.00	0.52 ± 0.24	
		GBT	0.75	0.82	0.78	0.87	0.79		0.71	0.72	0.78 ± 0.05	
	Mean ± SD		0.69 ± 0.22	0.72 ± 0.13	0.76 ± 0.17	0.80 ± 0.07	0.83 ± 0.10		0.81 ± 0.12	0.56 ± 0.41		
	AutoML	Vertex AI	0.81	0.89	0.93	--	0.84		0.92	0.84	0.87 ± 0.04	
		Azure	0.81	0.8	0.82	0.88	0.86		0.83	0.84	0.84 ± 0.02	
		Dataiku	0.77	0.82	0.83	0.93	0.87		0.84	0.75	0.83 ± 0.06	
	Mean ± SD		0.80 ± 0.02	0.85 ± 0.03	0.86 ± 0.05	0.91 ± 0.03	0.86 ± 0.01		0.86 ± 0.04	0.81 ± 0.04		
Recall	ML	RF	0.21	0.14	0.44	0.88	0.52		0.53	0.50	0.46 ± 0.22	
		SVM	0.08	0.34	0.14	0.26	0.49		0.53	0.00	0.26 ± 0.19	
		GBT	0.68	0.76	0.83	0.92	0.74		0.78	0.75	0.78 ± 0.07	
	Mean ± SD		0.32 ± 0.26	0.41 ± 0.26	0.47 ± 0.28	0.69 ± 0.30	0.58 ± 0.11		0.61 ± 0.12	0.42 ± 0.31		
	AutoML	Vertex AI	0.71	0.91	0.82	--	0.65		0.75	0.68	0.75 ± 0.08	
		Azure	0.68	0.76	0.75	0.76	0.72		0.73	0.68	0.73 ± 0.03	
		Dataiku	0.77	0.78	0.91	0.93	0.81		0.81	0.84	0.84 ± 0.06	
	Mean ± SD		0.72 ± 0.04	0.82 ± 0.07	0.83 ± 0.07	0.85 ± 0.09	0.73 ± 0.07		0.76 ± 0.03	0.73 ± 0.08

The improved performances of autoML-based nanotoxicity prediction models can be ascribed to their workflow, which differs from that of ML-based model development. As shown in Fig. 1, autoML follows more comprehensive steps of algorithm selection, and hyperparameter tunung to find the optimal combination.

**Fig. 3:**
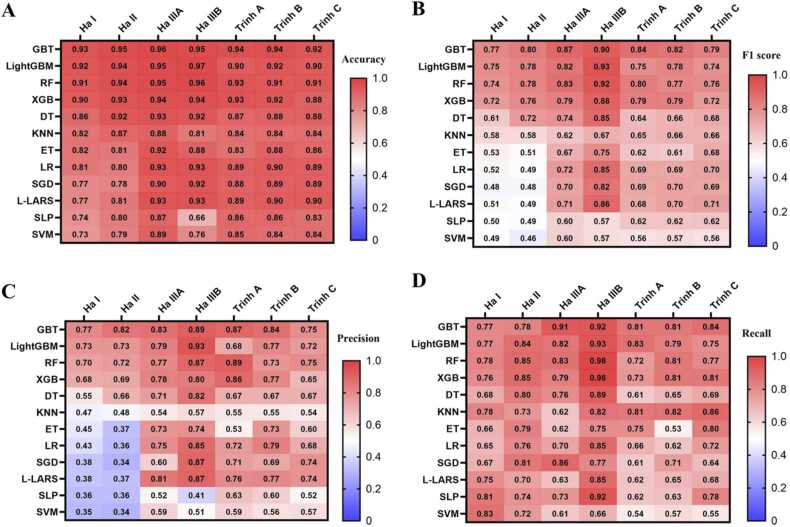
Accuracy (A), F1 score (B), precision (C), and recall (D) of models built from different algorithms (GBT: gradient boosted trees, LightGBM: light gradient boosted machine, RF: random forest, XGB: XGBoost, DT: decision tree, KNN: k-nearest neighbor, ET: extra trees, LR: logistic regression, SGD: stochastic gradient, L-LARS: LASSO-LARS, SLP: single layer perceptron, SVM: support vector machine) in Dataiku autoML platform.

Considering the large number of ML algorithms available for prediction model developement, one of the challenging task in nanotoxicity prediction model development is selecting the appropriate algorithm for a given dataset. AutoML platforms address this challenge via combinatorialy testing numerous algorithms and selecting the best ones based on the comparisons of their performances. For instance, in the Dataiku autoML platform, users can include the algorithms to be tested from a pool of algorithms and select the best performing algorithm based on comparisons of various performance measures. The heat maps in Fig. 3 compare accuracy, F1 score, precision, and recall values of oxide and metal datasets (i.e., Ha I, II, IIIA, IIIB, Trinh A, B, and C) for 12 different algorithms to demonstrate the algorithm selection procedure in autoML platform. We can clearly observe that gradient boosted trees (GBT) algorithm is the best performing algorithm for the datasets tested with the Dataiku autoML platform. This algorithm selection strategy was similarly adopted by Vertex AI and Azure platforms [44] and the selected algorithms for all the datasets and autoML platforms were listed in Table S2, along with the preprocessing methods. Additionally, overcoming the challenge of hyperparameter tuning to find the optimal combination is another critical aspect of developing nanotoxicity prediction models [45]. AutoML platforms overcome this challenging task via combinatorial testing hyperparameters and selecting the optimal conditions based on the comparisons of their performances. For example, as shown in Fig. S10, when Dataiku built a random forest model for the Ha I dataset, it tuned the maximum tree depth, maximum features, minimum samples per leaf, maximum number of trees, and minimum samples to split and returned performance measures against these hyperparameters. Other autoML platforms also work in similar ways, although their method for searching the hyperparameters can change from one platform to another [24].

### 3.3 Comparison of autoML platforms: Vertex AI vs. Azure vs. Dataiku

**Fig. 4:**
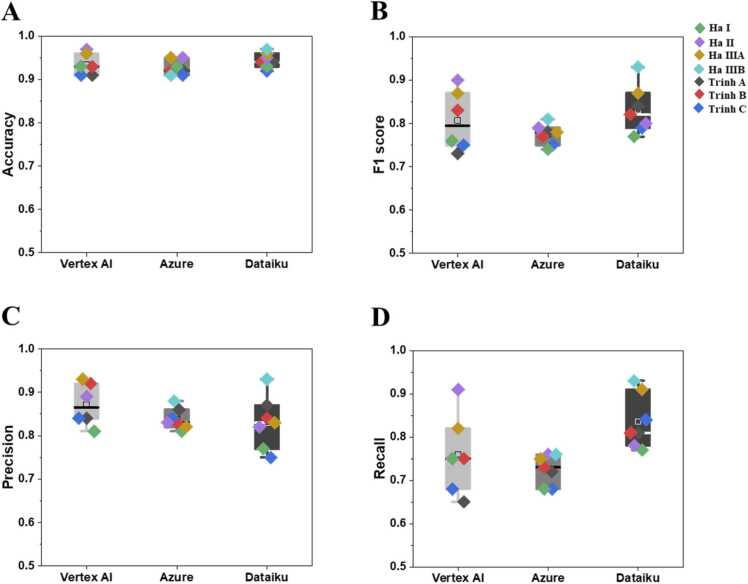
The comparison between different autoML platforms (Vertex AI, Azure, and Dataiku) in terms of accuracy (A), F1 score (B), precision (C), and recall (D). In the box plot each dot represents one dataset. The black line is the median and the square is the mean.

Comparisons of three autoML platforms (Vertex AI, Azure, and Dataiku) with respect to their performance measures are shown in Fig. 4. All three platforms displayed high accuracy with mean values of 0.93 – 0.94, while their F1 score, precision, and recall for each platform displayed slightly lower mean values of 0.77 – 0.83, 0.83 – 0.87, and 0.73 – 0.84, respectively (see Table 2). Although none of the three autoML platforms significantly outperformed the others, there are some distinctions between them in terms of the options available for choosing technical features throughout the model development steps. As previously mentioned, in consideration of users with limited or no background in machine learning (ML) and programming, we have deliberately selected three user-friendly autoML platforms for this study. Notably, these platforms have been chosen for their ability to operate without necessitating coding or programming expertise. However, despite the fact that all three autoML platforms tested in this study require only a few decisions over technical features, there are some differences among these autoML platforms, in terms of the available options for these technical features during the steps of data preprocessing, algorithm selection, and hyperparameter tuning. In the Dataiku and Azure platforms, users have more options to choose these technical features, while Vertex AI does not allow users to choose options for most technical features. For instance, the Dataiku and Azure platforms allow users to choose a normalization method among the options of z-score and min-max normalization, as well as the candidate algorithms included in the algorithm pool, while Vertex AI does not provide those options. In addition, the Dataiku platform also allow users to choose several technical features, such as type of hyperparameters for each algorithm, selection strategy (e.g., grid, random and bayes search), and criteria measures (e.g., F1 score, accuracy, AUC, log loss, cumulative fit, etc.) during the hyperparameter tuning step, while Azure and Vertex AI do not provide those options. Model interpretation was also provided by Dataiku platform, by listing the most important features and how they influenced the endpoint. In our results, the top three most important features are dose, enthalpy of formation, and cell line for Ha I dataset (Fig. S11A–D), and dose, time and cell line for Trinh A dataset (Fig. S11E–H). The interpretation provides useful information for better nanomaterial design, such as the proper dosage that doesn’t cause cell death for in vitro experiments ( < 50 μg/mL for metal oxide nanoparticles and < 25 μg/mL for metal nanoparticles), or the influence of different cell lines and assays on cell viability. Users can choose a platform that best suits their knowledge of predictive model development and its technical features. When users have some basic knowledge on technical terms in ML and want more control over the model development process, the Dataiku and Azure platform will be preferred, while the Vertex AI platform is recommended for those with limited understanding on ML algorithms and model development process. Additionally, all three autoML platforms tested in this study do not provide information on the applicability domain, where the models provide predictions with given reliability. Since the nanotoxicity prediction model is applicable to a new nanomaterial only if its properties fall within the applicability domain, the applicability domain of autoML based models needs to be calculated manually.

### 3.4 Effect of data quality and dataset size

**Fig. 5:**
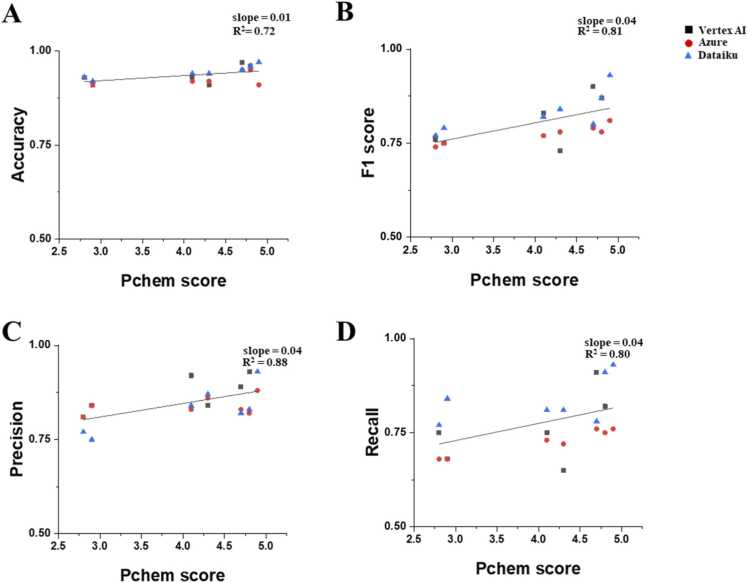
The effect of data quality (PChem score) on model performance of three different autoML models such as Vertex AI, Azure, and Dataiku, measured by accuracy (A), F1 score (B), precision (C), and recall (D).

As described in Table 1, the datasets used in this study were originally prepared to test the effects of different data quality (i.e., PChem score) and dataset size (i.e., numbers of rows) [11], [12]. In Fig. 5, the performance values of all autoML-based models built on the seven datasets were plotted to examine the effect of data quality. Overall, as data quality improved, all four performance measures exhibited increasing trends. The accuracy, F1 score, precision, and recall values increased with the slope of 0.01, 0.04, 0.04 and 0.04, respectively, as the data quality increased. As with previous comparisons, the F1 score, precision, and recall values were found to be more sensitive to data quality, while the accuracy value was found to be less responsive to the quality of data. These trends agree well with those of conventional ML-based models (i.e., RF, GBT, and SVM) presented in Fig. S12 as well as earlier studies on the effect of data quality on nanotoxicity models’ performance [11], [12]. These findings confirm and emphasize the importance of data quality in the development of predictive models, regardless of the chosen algorithms and platforms.

**Fig. 6:**
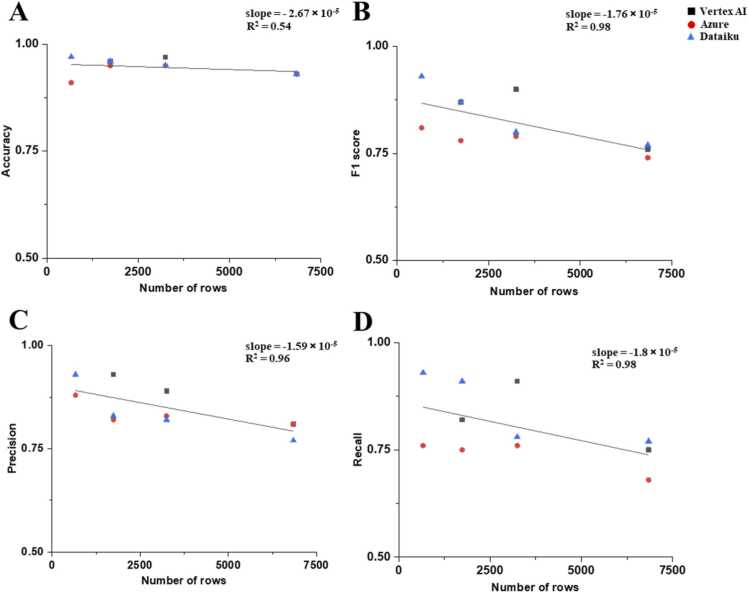
The effect of dataset size (number of rows) on model performance of autoML models such as Vertex AI, Azure, and Dataiku, measured by accuracy (A), F1 score (B), precision (C), and recall (D).

The effect of dataset size on the performance of autoML-based models was also tested and is presented in Fig. 6. In this analysis, we used the dataset Ha I, II, IIIA, and IIIB, each having different numbers of rows and PChem scores, as described in Table 1. Interestingly, the model performance measures displayed decrements as the size of the dataset increases. As the number of rows in the dataset increased from 666 to 6842, the accuracy, F1 score, precision, and recall values were decreased with the slopes of - 2.67 × 10^-5^, - 1.76 × 10^-5^, - 1.59 × 10^-5^, and - 1.80 × 10^-5^, respectively. Similar trends were observed for those of conventional ML-based models (i.e., RF, GBT, and SVM) shown in Fig. S13. However, these decreasing trends seem mostly due to the effect of data quality rather than dataset size, since the quality of these oxide datasets increases with decreasing dataset size. It is assumed that the size of these datasets has already reached the saturation point where sufficiently accurate learning is feasible [46], and that data quality, rather than the size of the datasets, becomes a dominant factors. This suggests that the dataset size is not a limiting factor for the performance of autoML-based models for the datasets used in this study.

## 4 Conclusions

In this study, nanotoxicity prediction models were developed with three autoML platforms (i.e., Vertex AI, Azure, and Dataiku) and seven publicly available datasets for oxide and metal nanomaterials (i.e., Ha I, II, IIIA, IIIB, Trinh A, B, and C). The performance of these autoML-based models (i.e., accuracy, F1 score, precision, and recall) was then compared to those of conventional ML-based models, which clearly demonstrated that the autoML platforms are able to produce more reliable nanotoxicity prediction models performing better than those built with conventional ML algorithms. While none of the three autoML platforms outperformed the others significantly, there are some distinctions between these autoML platforms in terms of the options for choosing technical features throughout the model development steps. Users can choose a platform that best suits their knowledge of predictive model development and its technical features. Additionally, prediction models constructed from better quality datasets (i.e., those with higher physicochemical scores) performed better than those constructed from poorer quality datasets, indicating that future studies with higher-quality datasets can further improve the performance of those autoML-based nanotoxicity prediction models. Although we demonstrated improved model performance using previously published and publicly available datasets, additional follow-up study is necessary, including validation studies using larger, higher-quality datasets, as well as the adoption of novel autoML platforms utilizing new artificial intelligence (AI) technology, which will aid in broadening applicability domains and improving model performance.

## CRediT authorship contribution statement

**Xiao Xiao**: Writing – original draft, Writing – review & editing, Software, Visualization, Investigation. **Tung X. Trinh**: Writing – original draft, Validation, Visualization, Investigation. **Zayakhuu Gerelkhuu**: Writing - review & editing, Validation, Visualization. **Eunyong Ha**: Validation, Writing – review & editing. **Tae Hyun Yoon**: Conceptualization, Methodology, Writing – review & editing, Supervision.

## Declaration of Competing Interest

The authors declare that they have no known competing financial interests or personal relationships that could have appeared to influence the work reported in this paper.
