# Supplementary material for: Automated machine learning in nanotoxicity assessment: A comparative study of predictive model performance
Source: Comput Struct Biotechnol J. 2024 Feb 9;25:9–19. doi: 10.1016/j.csbj.2024.02.003 (PMC10899003; doi:10.1016/j.csbj.2024.02.003)
Supplement: Supplementary file 1 — Supplementary material [file mmc1.docx]

**Automated Machine Learning in Nanotoxicity Assessment: A Comparative Study of Predictive Model Performance**

**Xiao Xiao^a^**^†^**, Tung X. Trinh^a^**^†^**, Zayakhuu Gerelkhuu^b,c^, Eunyong Ha^a^, and Tae Hyun Yoon^a,b,c*^**

^a^Department of Chemistry, College of Natural Sciences, Hanyang University, Seoul 04763, Republic of Korea.

^b^Institute of Next Generation Material Design, Hanyang University, Seoul 04763, Republic of Korea.

^c^Yoon Idea Lab. Co. Ltd, Seoul 04763, Republic of Korea

^†^These authors contributed equally to this work.

*Corresponding Author: Tae-Hyun Yoon

E-mail: [taeyoon@hanyang.ac.kr](mailto:taeyoon@hanyang.ac.kr)

Phone: +82-(0)2-2220-4593

Supplementary material

List of Figures and Tables

Figure S1. Dataset description of Ha I. (A) PChem properties of MeOx NP, (B) Dose-viability data of MeOx NP, and (C) Cell species and MeOx types.

Figure S2. Dataset description of Ha II. (A) PChem properties of MeOx NP, (B) Dose-viability data of MeOx NP, and (C) Cell species and MeOx types.

Figure S3. Dataset description of Ha IIIA. (A) PChem properties of MeOx NP, (B) Dose-viability data of MeOx NP, and (C) Cell species and MeOx types.

Figure S4. Dataset description of Ha IIIB. (A) PChem properties of MeOx NP, (B) Dose-viability data of MeOx NP, and (C) Cell species and MeOx types.

Figure S5. Dataset description of Trinh A. (A) PChem properties of metal NP, (B) Dose-viability data of metal NP, and (C) Cell species and metal types.

Figure S6. Dataset description of Trinh B. (A) PChem properties of metal NP, (B) Dose-viability data of metal NP, and (C) Cell species and metal types.

Figure S7. Dataset description of Trinh C. (A) PChem properties of metal NP, (B) Dose-viability data of metal NP, and (C) Cell species and metal types.

Figure S8. Assessment of activity cliffs for Ha I and Trinh A datasets via Banerjee and Roy Similarity Coefficients 1 and 2 (S_m_^1^ and S_m_^2^). (A) S_m_^1^ for Ha I test set, (B) S_m_^1^ for Trinh A test set, (C) S_m_^2^ for Ha I test set, and (D) S_m_^2^ for Trinh A test set.

Figure S9. Accuracy (A), F1 score (B), precision (C), and recall (D) of ML and autoML models on datasets Ha I, II, IIIA, and IIIB, and Trinh A, B, C when the training and test sets are the same for a particular dataset. In the boxplot each dot represents one algorithm. RF: random forest. SVM: support vector machine. GBT: gradient boosted trees.

Figure S10. Hyperparameter tuning in Dataiku platform

Figure S11. Model interpretation provided by Dataiku platform. (A) Most important features in the model built with Ha I dataset. (B-D) The influence of top 3 features (dose, enthalpy of formation, and cell line) on the toxicity endpoint for Ha I dataset. (E) Most important features in the model built with Trinh A dataset. (F-H) The influence of top 3 features (dose, time, and cell line) on the toxicity endpoint for Trinh A dataset.

Figure S12. Effect of quality (PChem score) on model performance of ML models, measured by accuracy (A), F1 score (B), precision (C), and recall (D).

Figure S13. Effect of dataset size (number of rows) on model performance of ML models, measured by accuracy (A), F1 score (B), precision (C), and recall (D).

Tabel S1. Data preprocessing method by different autoML platforms

Tabel S2. Algorithms chosen by different autoML platforms


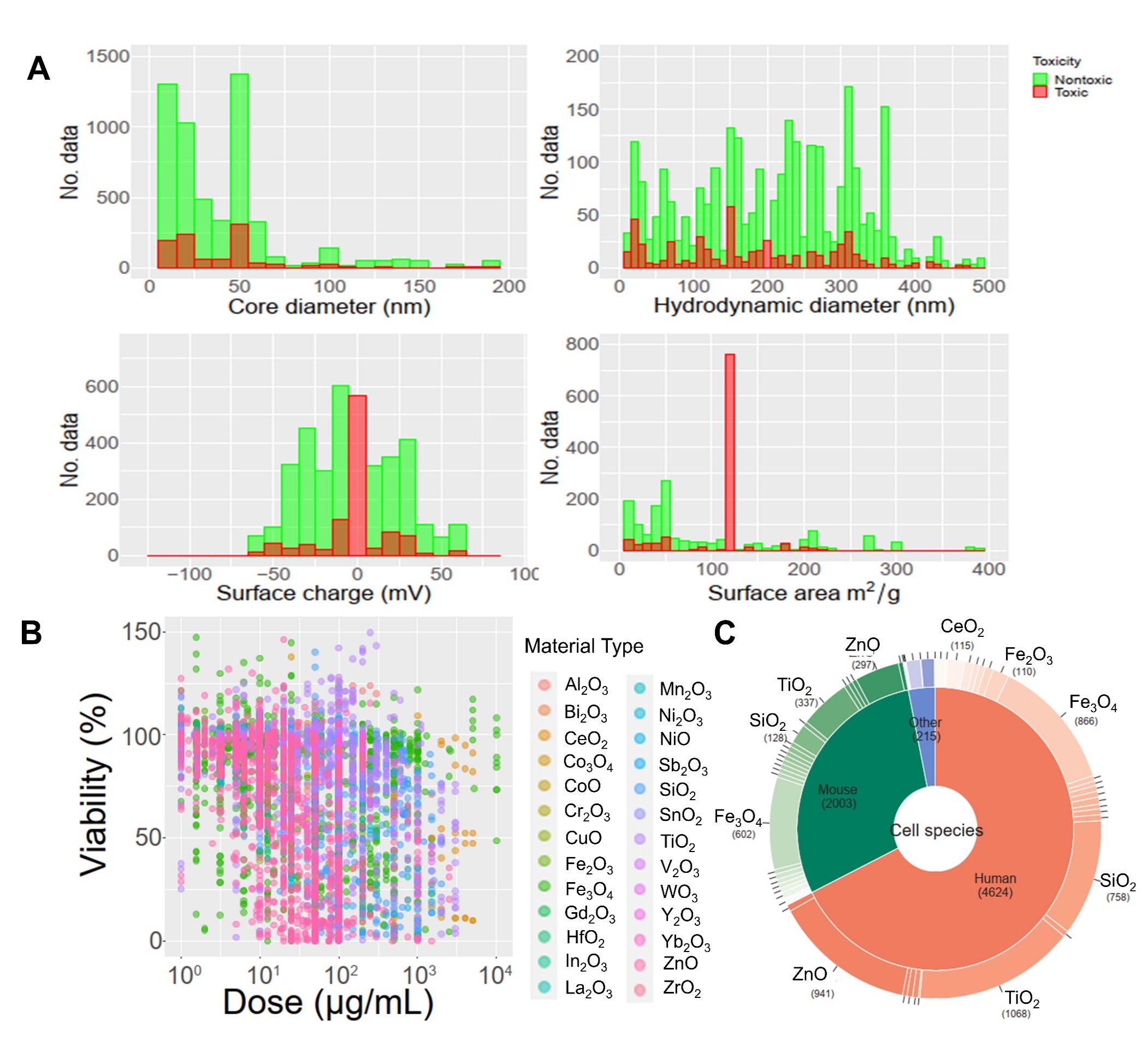


Figure S1. Dataset description of Ha I. (A) PChem properties of MeOx NP, (B) Dose-viability data of MeOx NP, and (C) Cell species and MeOx types.


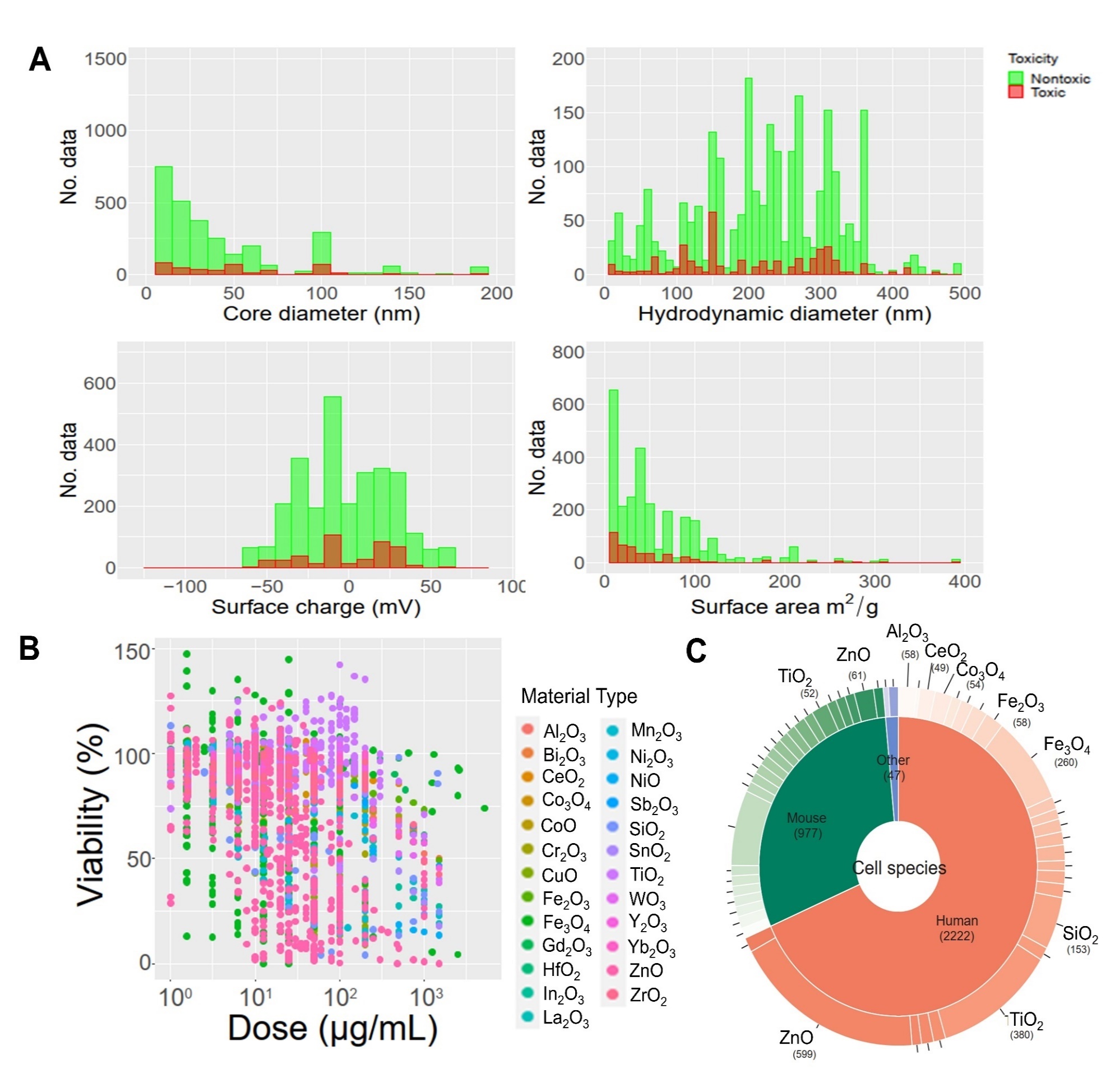


Figure S2. Dataset description of Ha II. (A) PChem properties of MeOx NP, (B) Dose-viability data of MeOx NP, and (C) Cell species and MeOx types.


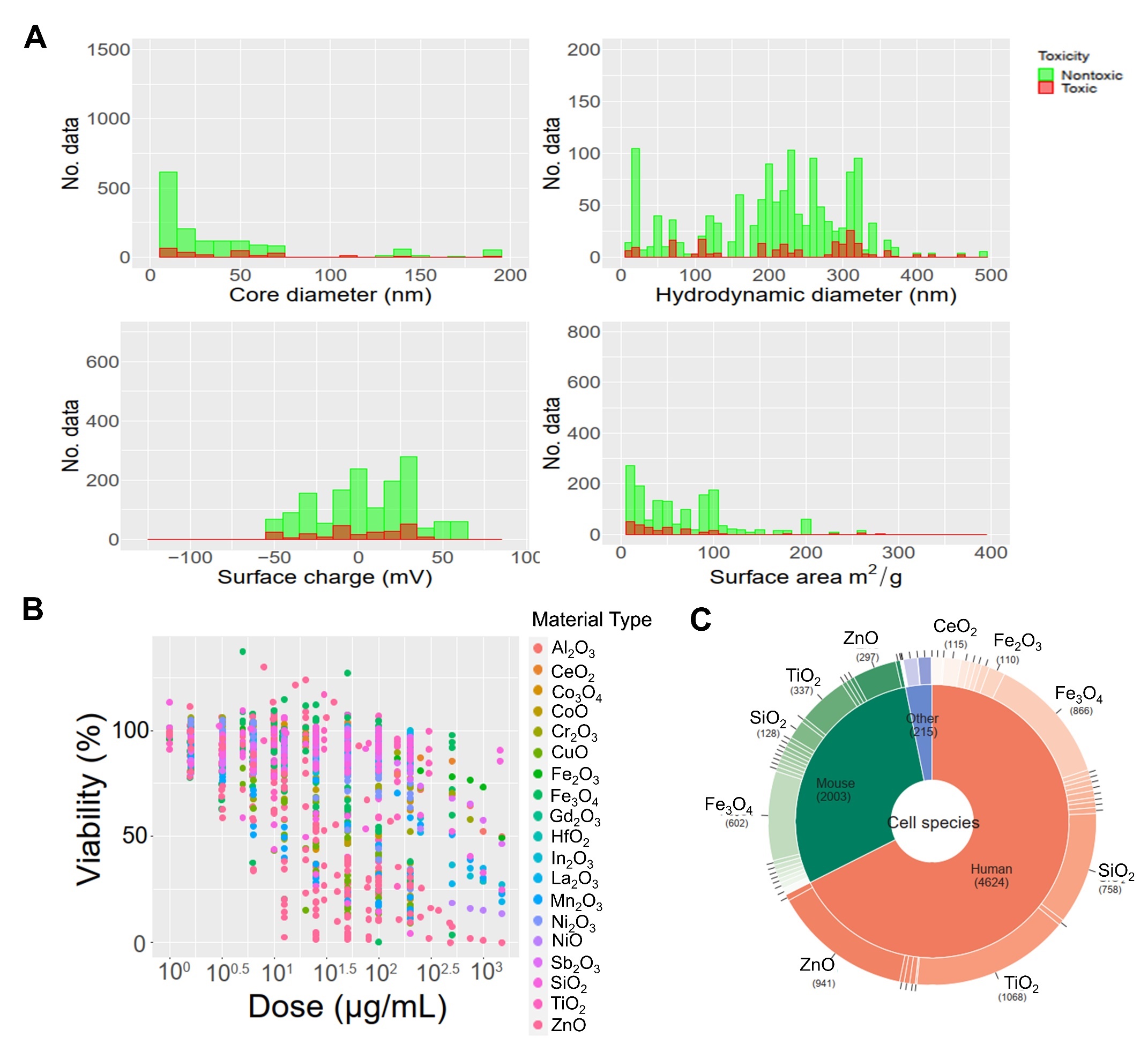


Figure S3. Dataset description of Ha IIIA. (A) PChem properties of MeOx NP, (B) Dose-viability data of MeOx NP, and (C) Cell species and MeOx types.


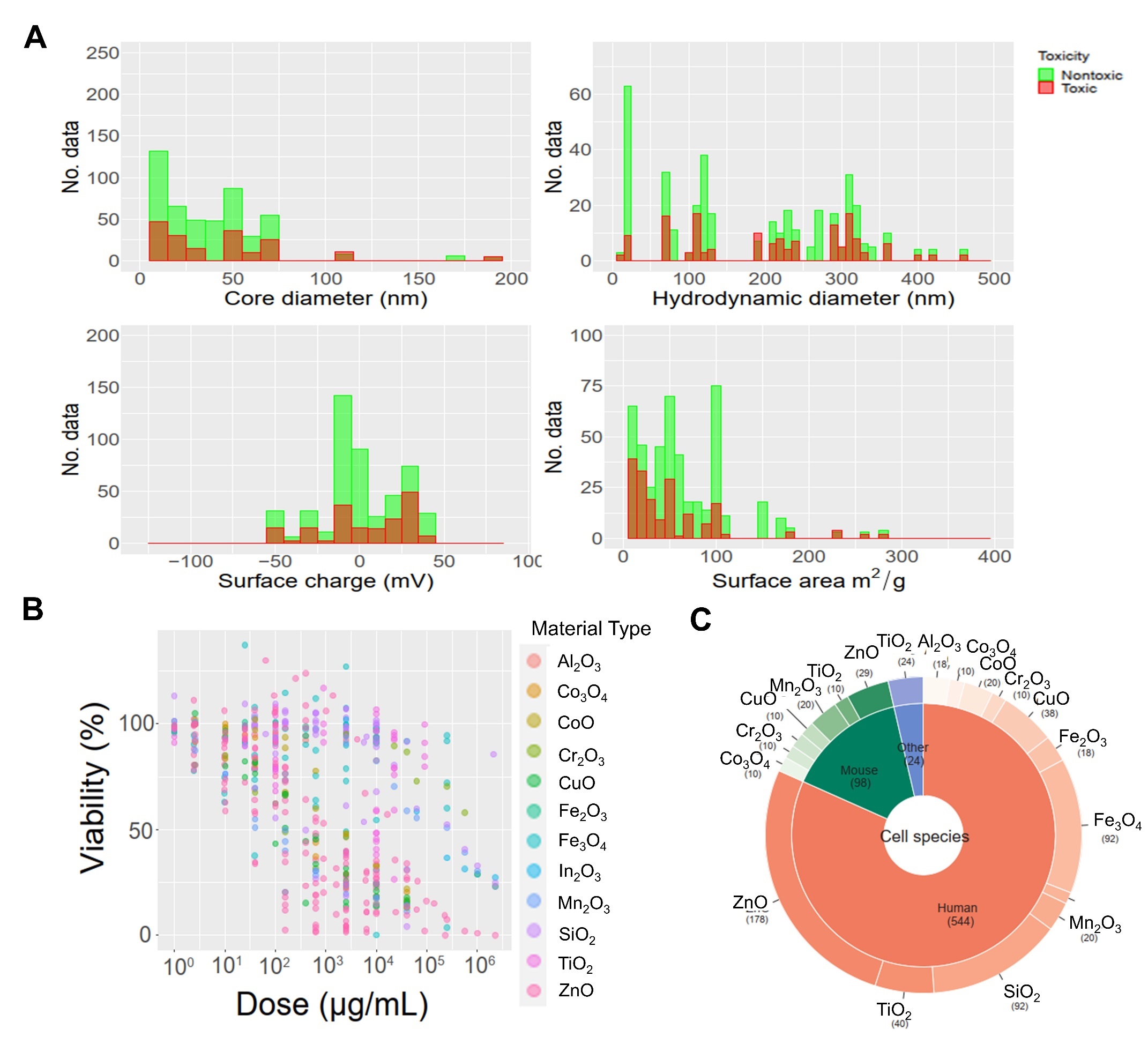


Figure S4. Dataset description of Ha IIIB. (A) PChem properties of MeOx NP, (B) Dose-viability data of MeOx NP, and (C) Cell species and MeOx types.


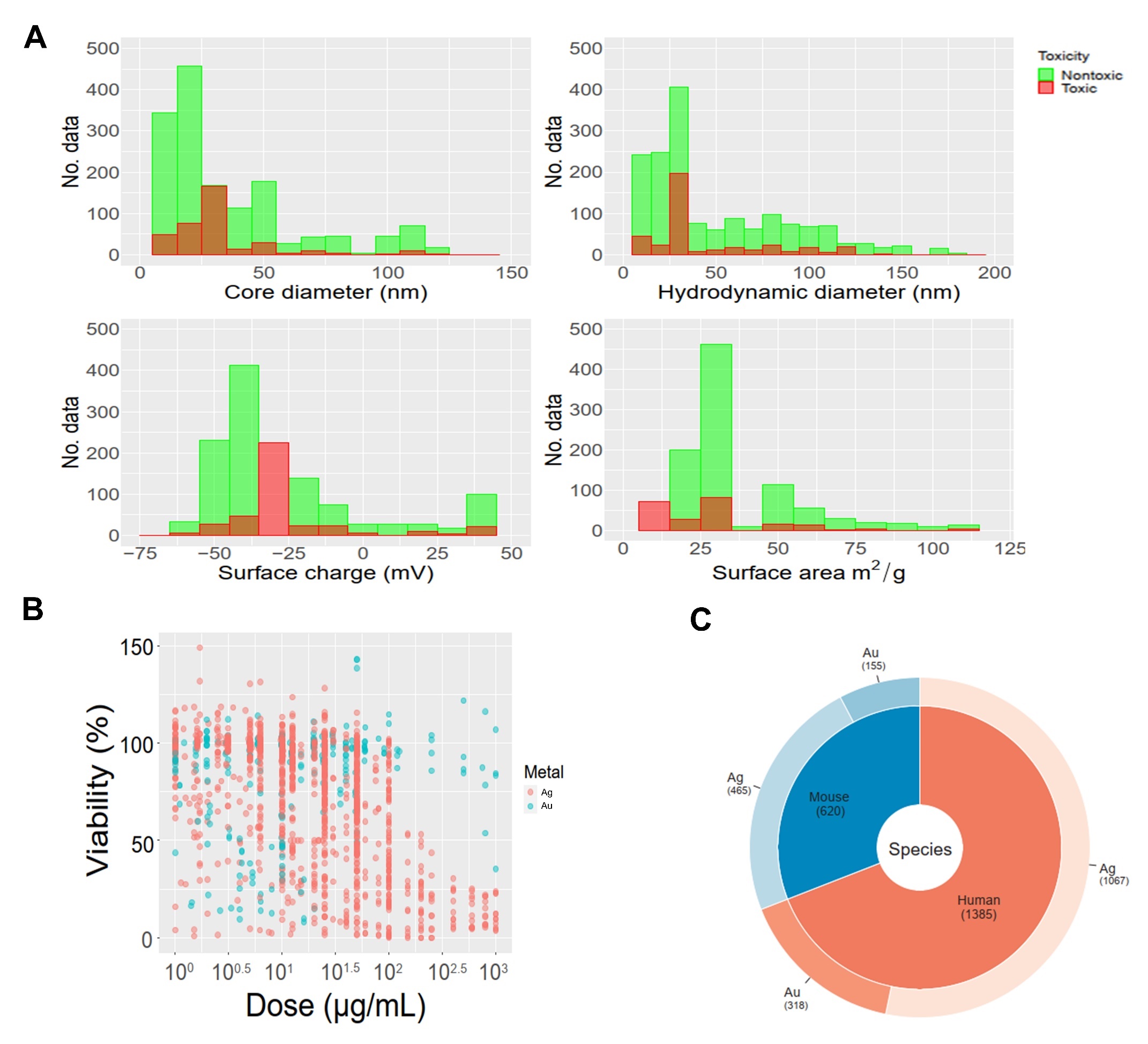


Figure S5. Dataset description of Trinh A. (A) PChem properties of metal NP, (B) Dose-viability data of metal NP, and (C) Cell species and metal types.


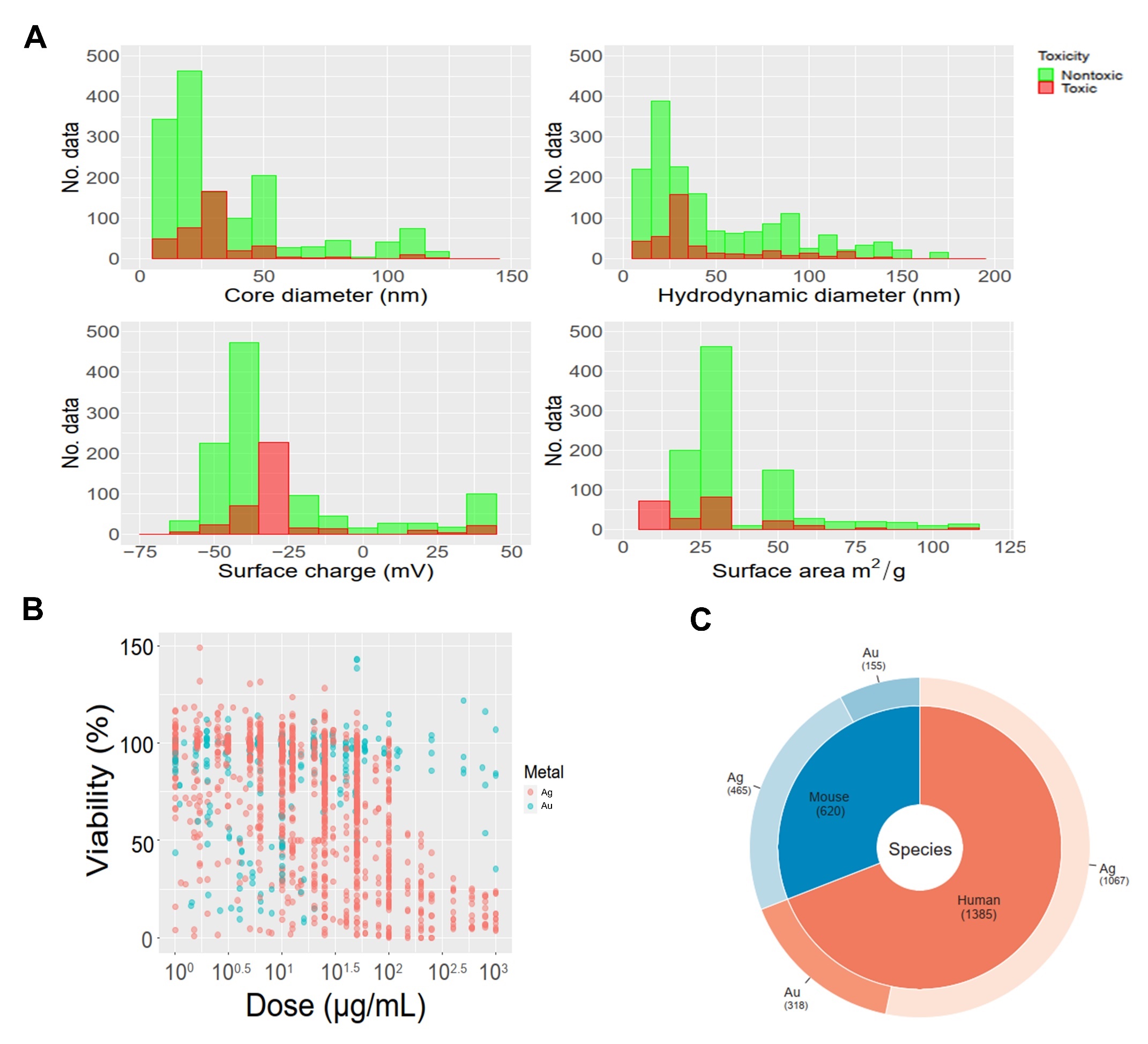


Figure S6. Dataset description of Trinh B. (A) PChem properties of metal NP, (B) Dose-viability data of metal NP, and (C) Cell species and metal types.


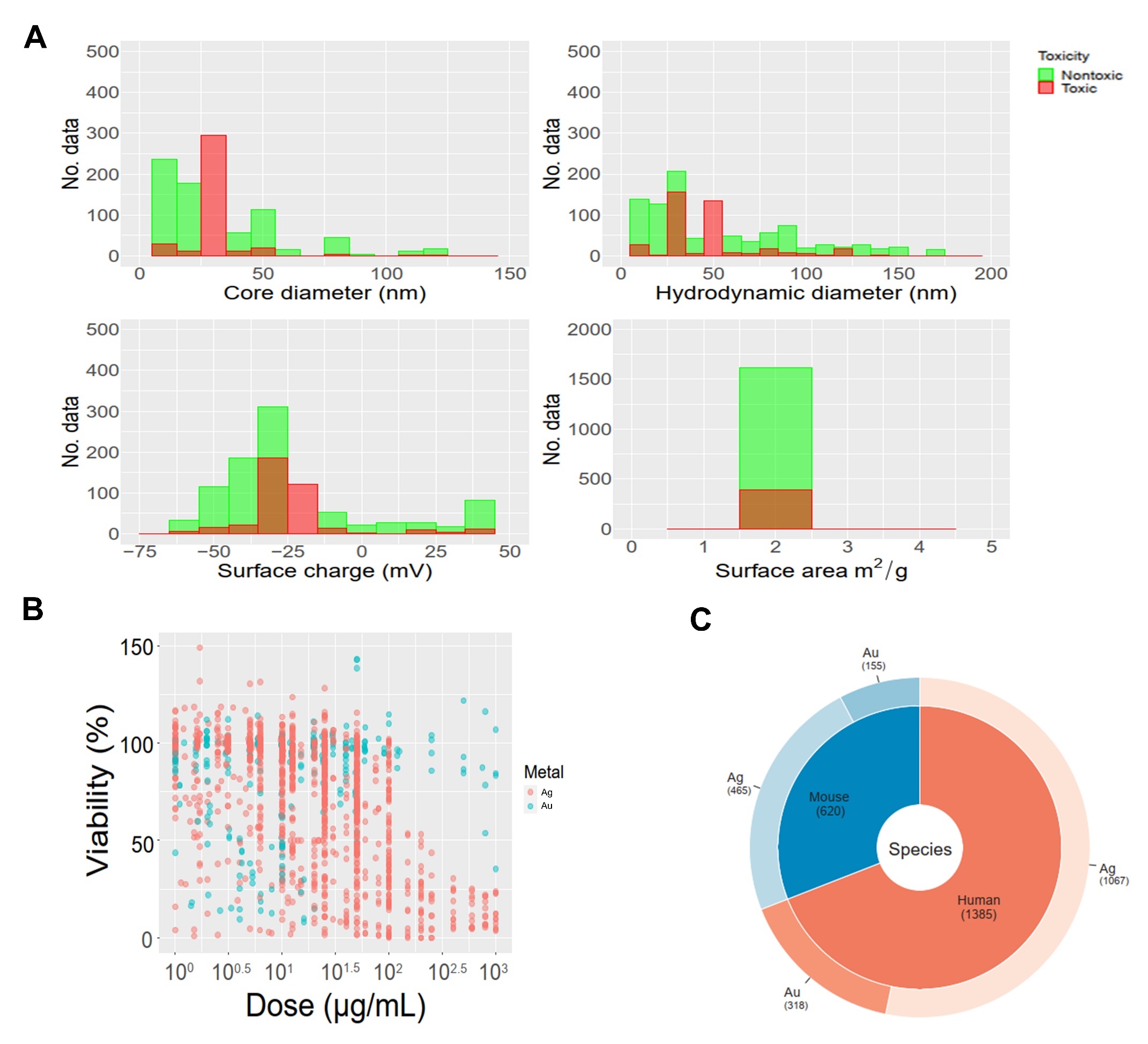


Figure S7. Dataset description of Trinh C. (A) PChem properties of metal NP, (B) Dose-viability data of metal NP, and (C) Cell species and metal types.


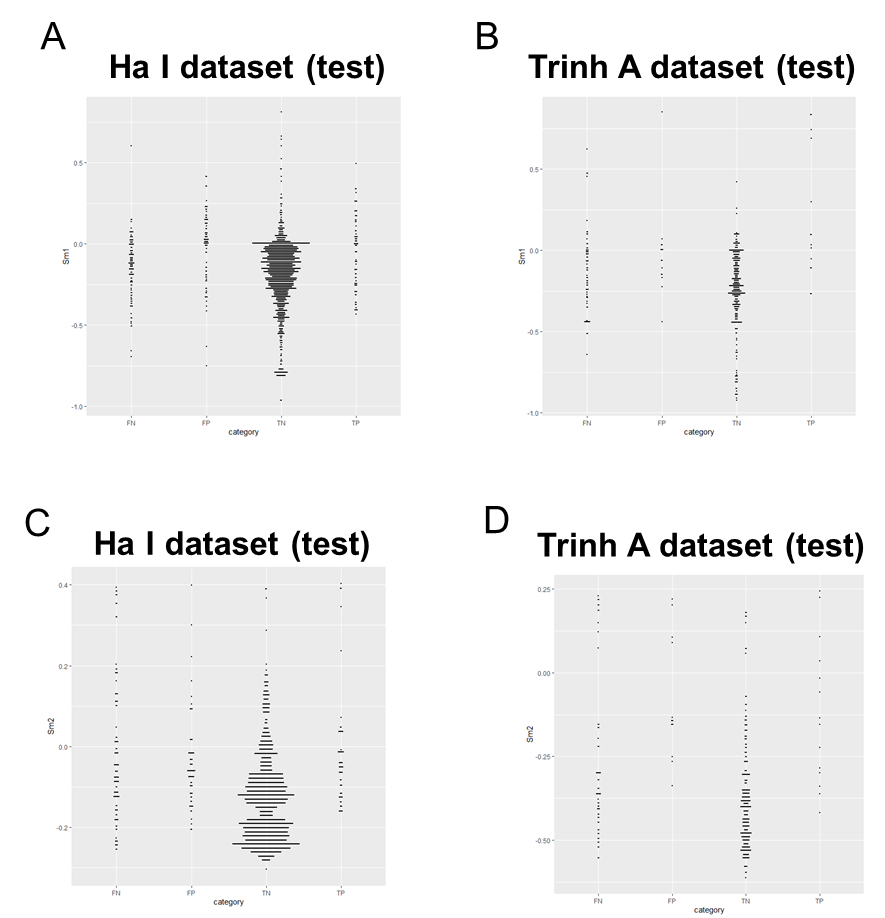


Figure S8. Assessment of activity cliffs for Ha I and Trinh A datasets via Banerjee and Roy Similarity Coefficients 1 and 2 (S_m_^1^ and S_m_^2^). (A) S_m_^1^ for Ha I test set, (B) S_m_^1^ for Trinh A test set, (C) S_m_^2^ for Ha I test set, and (D) S_m_^2^ for Trinh A test set.


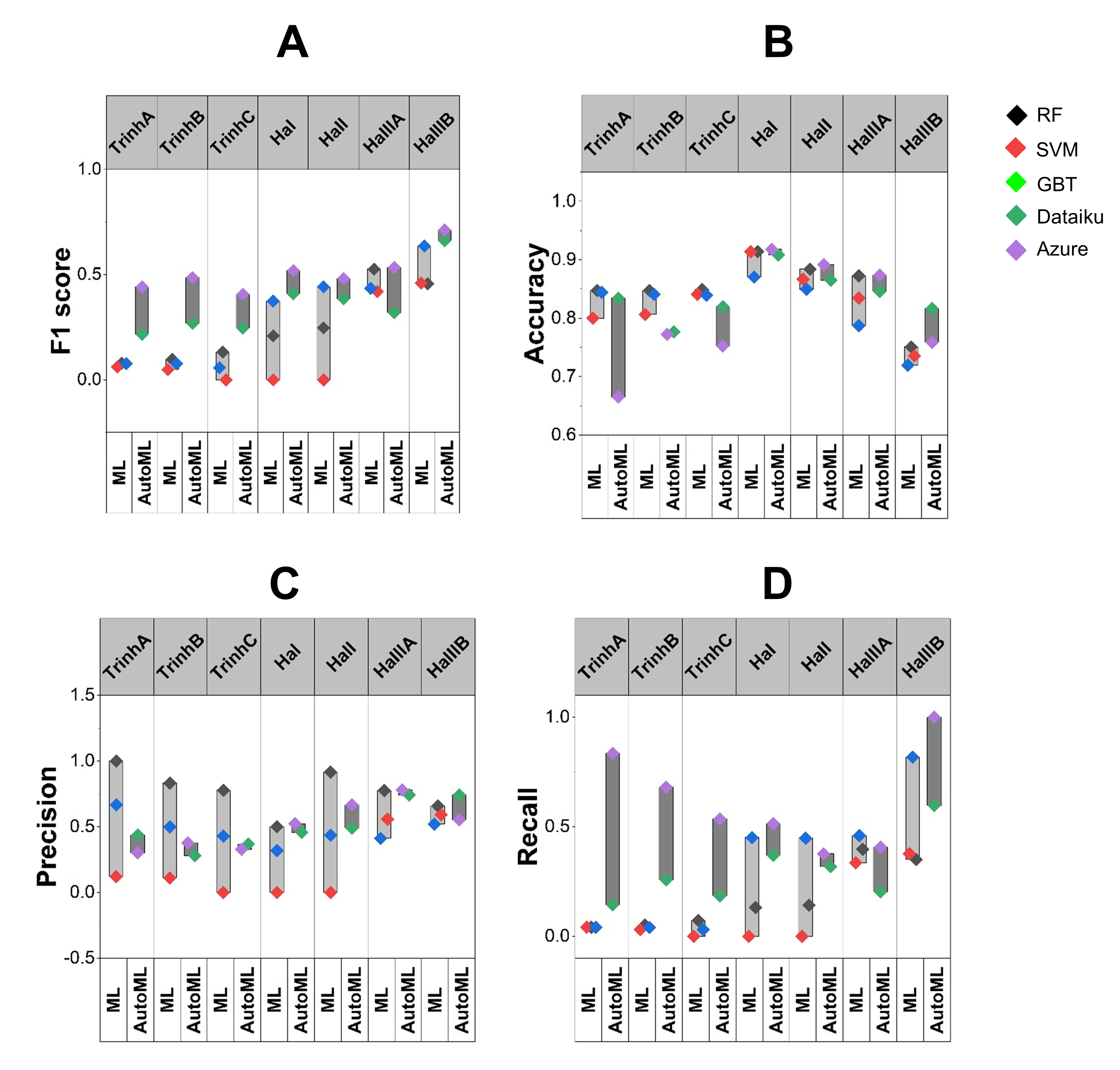


Figure S9. Accuracy (A), F1 score (B), precision (C), and recall (D) of ML and autoML models on datasets Ha I, II, IIIA, and IIIB, and Trinh A, B, C when the training and test sets are the same for a particular dataset. In the boxplot each dot represents one algorithm. RF: random forest. SVM: support vector machine. GBT: gradient boosted trees.


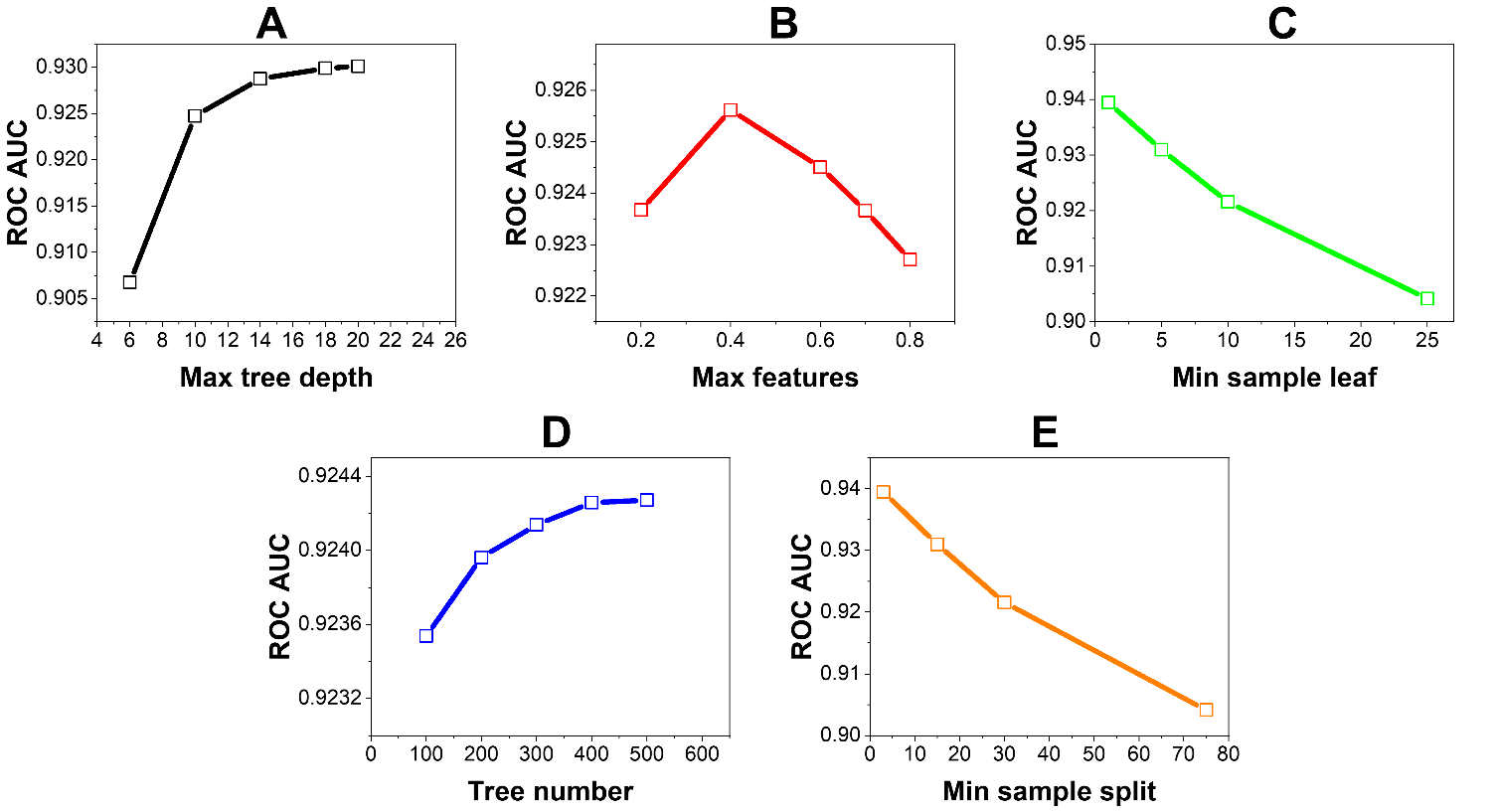


Figure S 10. Hyperparameter tuning in Dataiku platform


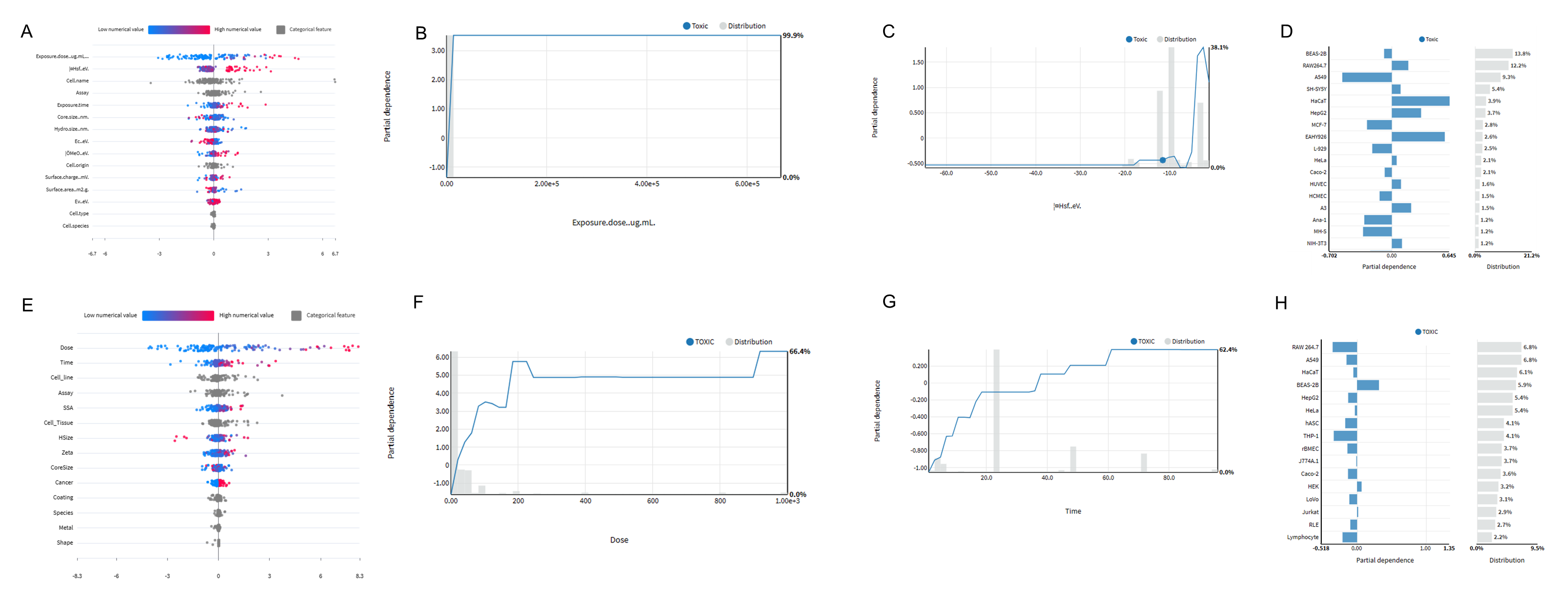


Figure S11. Model interpretation provided by Dataiku platform. (A) Most important features in the model built with Ha I dataset. (B-D) The influence of top 3 features (dose, enthalpy of formation, and cell line) on the toxicity endpoint for Ha I dataset. (E) Most important features in the model built with Trinh A dataset. (F-H) The influence of top 3 features (dose, time, and cell line) on the toxicity endpoint for Trinh A dataset.


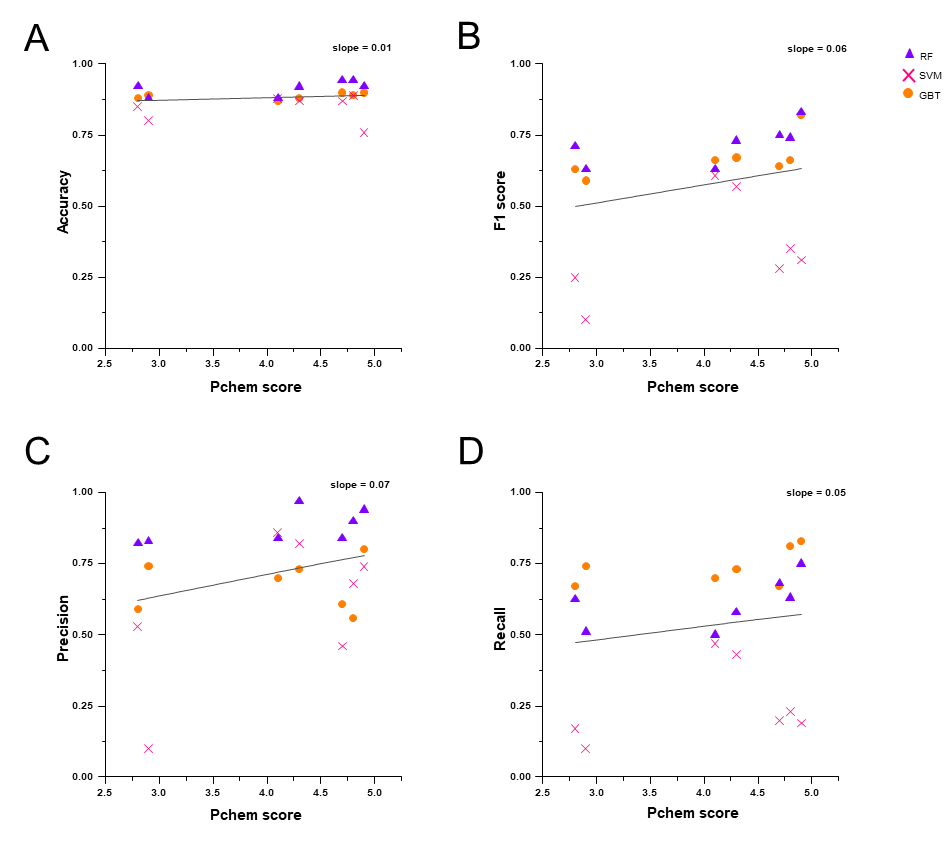


Figure S12. Effect of quality (PChem score) on model performance of ML models, measured by accuracy (A), F1 score (B), precision (C), and recall (D).


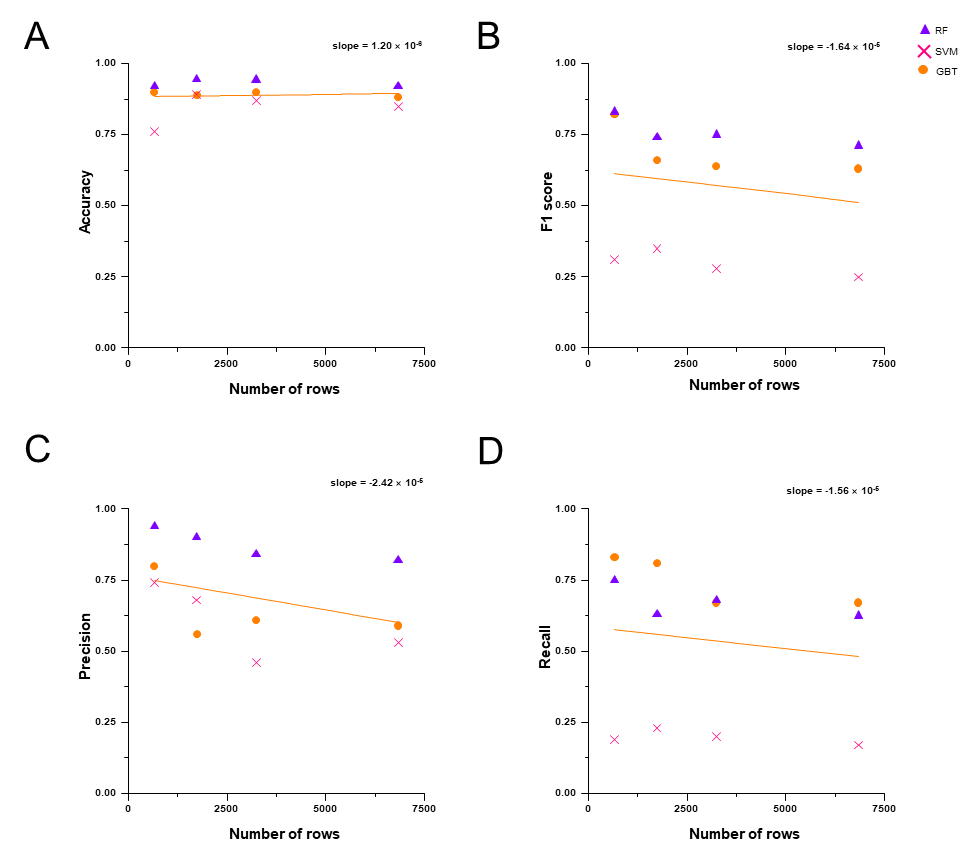


Figure S13. Effect of dataset size (number of rows) on model performance of ML models, measured by accuracy (A), F1 score (B), precision (C), and recall (D).

Tabel S 1. Examples of some well-known nanomaterials in this study’s datasets. Abbreviations for N is for non-toxic and T is for toxic.

| Reference | Descriptors | | | | | | | | | | Experimental toxicity | Predicted toxicity | | | | | |
| --- | --- | --- | --- | --- | --- | --- | --- | --- | --- | --- | --- | --- | --- | --- | --- | --- | --- |
|  | Material type | Core size (nm) | Hydrodynamic size (nm) | Surface charge (mV) | Surface area (m^2^/g) | Assay | Cell name | Exposure time (h) | Exposure dose (μg/mL) | Cell type, Cell origin, ΔHsf (eV),... |  | RF | SVM | GBT | Vertex AI | Azure | Dataiku |
| [S1] | Al_2_O_3_ | 14.7 | 260.4 | 0 | 117 | MTS | BEAS-2B | 24 | 0.4 | … | N | N | N | N | N | N | N |
| [S1] | CeO_2_ | 18.3 | 261.6 | 21.4 | 117 | MTS | BEAS-2B | 24 | 0.4 | … | N | N | N | N | N | N | N |
| [S2] | Fe_2_O_3_ | 46.7 | 415 | -2.9 | 117 | WST | ECV-304 | 24 | 35 | … | N | N | N | T | N | N | N |
| [S1] | TiO_2_ | 12.6 | 109.2 | -19.4 | 117 | MTS | BEAS-2B | 24 | 0.4 | … | N | N | N | N | N | N | N |
| [S3] | ZnO | 23.47 | 415 | -2.9 | 117 | MTT | Human dermal fibroblast | 24 | 100 | … | T | N | N | T | T | T | T |
| [S4] | ZnO | 14.7 | 153 | -17.3 | 117 | MTT | HMDM | 24 | 50 | … | T | N | N | T | N | T | T |
| [S5] | Au | 17 | 36 | -37.5 | 18.29 | MTT | A549 | 24 | 15 | … | N | N | N | N | N | N | N |
| [S6] | Ag | 26 | 8.9 | -33.5 | 22 | MTT | Peritoneal exudates cells | 24 | 15 | … | T | N | N | N | T | N | T |

[S1] H. Zhang, Z. Ji, T. Xia, H. Meng, C. Low-Kam, R. Liu, S. Pokhrel, S. Lin, X. Wang, Y.P. Liao, M. Wang, L. Li, R. Rallo, R. Damoiseaux, D. Telesca, L. Mädler, Y. Cohen, J.I. Zink, A.E. Nel, Use of metal oxide nanoparticle band gap to develop a predictive paradigm for oxidative stress and acute pulmonary inflammation, ACS Nano. 6 (2012) 4349–4368. https://doi.org/10.1021/nn3010087.

[S2] F.Y. Yang, M.X. Yu, Q. Zhou, W.L. Chen, P. Gao, Z. Huang, Effects of iron oxide nanoparticle labeling on human endothelial cells, Cell Transplant. 21 (2012) 1805–1820. https://doi.org/10.3727/096368912X652986.

[S3] K. Meyer, P. Rajanahalli, M. Ahamed, J.J. Rowe, Y. Hong, ZnO nanoparticles induce apoptosis in human dermal fibroblasts via p53 and p38 pathways, Toxicol. Vitr. 25 (2011) 1721–1726. https://doi.org/10.1016/j.tiv.2011.08.011.

[S4] S. Tuomela, R. Autio, T. Buerki-Thurnherr, O. Arslan, A. Kunzmann, B. Andersson-Willman, P. Wick, S. Mathur, A. Scheynius, H.F. Krug, B. Fadeel, R. Lahesmaa, Gene Expression Profiling of Immune-Competent Human Cells Exposed to Engineered Zinc Oxide or Titanium Dioxide Nanoparticles, PLoS One. 8 (2013). https://doi.org/10.1371/journal.pone.0068415.

[S5] S.Y. Choi, S. Jeong, S.H. Jang, J. Park, J.H. Park, K.S. Ock, S.Y. Lee, S.W. Joo, In vitro toxicity of serum protein-adsorbed citrate-reduced gold nanoparticles in human lung adenocarcinoma cells, Toxicol. Vitr. 26 (2012) 229–237. https://doi.org/10.1016/j.tiv.2011.11.016.

[S6] Z. Shavandi, T. Ghazanfari, K.N. Moghaddam, In vitro toxicity of silver nanoparticles on murine peritoneal macrophages, Immunopharmacol. Immunotoxicol. 33 (2011) 135–140. https://doi.org/10.3109/08923973.2010.487489.

Tabel S2. Data preprocessing method and algorithms used in different autoML platforms

|  | Datasets | Vertex AI | Azure | Dataiku |
| --- | --- | --- | --- | --- |
| Preprocessing | Ha I | Not visible | Standardization; Label encoding | Standardization; Dummy encoding |
|  | Ha II |  | Standardization; Label encoding | Standardization; Dummy encoding |
|  | Ha IIIA |  | Standardization; Label encoding | Standardization; Dummy encoding |
|  | Ha IIB |  | Standardization; Label encoding | Standardization; Dummy encoding |
|  | Trinh A |  | MaxAbsScaler; Label encoding | Standardization; Dummy encoding |
|  | Trinh B |  | Standardization; Label encoding | Standardization; Dummy encoding |
|  | Trinh C |  | Standardization; Label encoding | Standardization; Dummy encoding |
| Algorithm selection | Ha I | Boosted Trees | XGBoost | Gradient Boosted Trees |
|  | Ha II | Neural network | XGBoost | Gradient Boosted Trees |
|  | Ha IIIA | Boosted Trees | XGBoost | Gradient Boosted Trees |
|  | Ha IIB | Not applicable (row number < 1000) | XGBoost | Gradient Boosted Trees |
|  | Trinh A | Neural network | LightGBM | Gradient Boosted Trees |
|  | Trinh B | Neural network | XGBoost | Gradient Boosted Trees |
|  | Trinh C | Neural network | XGBoost | Gradient Boosted Trees |

Standardization: rescales a numerical feature to have a mean of 0 and a standard deviation of 1; Label encoding: converting each categorical value in a column to a number; MaxAbsScaler: Scale each feature by its maximum absolute value; Dummy encoding: creates a vector of 0/1 flags of length equal to the number of categories in the categorical variable.
